# Comprehensive antifungal investigation of green synthesized silver nanoformulation against four agriculturally significant fungi and its cytotoxic applications

**DOI:** 10.1038/s41598-024-56619-9

**Published:** 2024-03-11

**Authors:** Jyoti Singh, Ankit Kumar, Amit Singh Nayal, Sagar Vikal, Gyanika Shukla, Amardeep Singh, Anupma Singh, Sakshi Goswami, Ashwani Kumar, Yogendra K. Gautam, Yeshvandra Verma, Shailendra Singh Gaurav, Dharmendra Pratap

**Affiliations:** 1 Plant Molecular Virology Laboratory, Department of Genetics and Plant Breeding, Chaudhary Charan Singh University, Meerut, Uttar Pradesh 250004 India; 2 Department of Statistics, Chaudhary Charan Singh University, Meerut, Uttar Pradesh 250004 India; 3 Smart Materials and Sensor Laboratory, Department of Physics, Chaudhary Charan Singh University, Meerut, 250004 Uttar Pradesh India; 4 NanoScience and NanoBiology Laboratory, Department of Genetics and Plant Breeding, Chaudhary Charan Singh University, Meerut, Uttar Pradesh 250004 India; 5 Department of Zoology, Chaudhary Charan Singh University, Meerut, Uttar Pradesh 250004 India; 6 Department of Toxicology, Chaudhary Charan Singh University, Meerut, Uttar Pradesh 250004 India; 7 Departemnt of Physics, Regional Institute of Education (RIE), Bhubaneswar, Odisha 751022 India

**Keywords:** Green synthesis, Silver nanoparticles, Polyethylene glycol, *Colletotrichum falcatum*, Antifungal, Cytotoxicity, Biotechnology, Microbiology, Nanoscience and technology

## Abstract

The present study reports the green synthesis of silver nanoparticles (AgNPs) in powder form using the leaf extract of *Azadirachta indica*. The synthesis of AgNPs was confirmed by UV–vis spectroscopy, FTIR, XRD, FESEM, and EDX. The synthesized AgNPs were in a powdered state and dispersed completely in 5% polyethylene glycol (PEG) and demonstrated prolonged shelf life and enhanced bioavailability over a year without any aggregation. The resulting silver nanoformulation demonstrated complete inhibition against *Sclerotinia sclerotiorum* and *Colletotrichum falcatum* and 68% to 80% inhibition against *Colletotrichum gloeosporioides* and *Rhizoctonia solani* respectively, at 2000 ppm. The EC_50_ values determined through a statistical analysis were 66.42, 157.7, 19.06, and 33.30 ppm for *S. sclerotiorum*, *C. falcatum*, *C. gloeosporioides*, and *R. solani* respectively. The silver nanoformulation also established significant cytotoxicity, with a 74.96% inhibition rate against the human glioblastoma cell line U87MG at 250 ppm. The IC_50_ value for the cancerous cell lines was determined to be 56.87 ppm through statistical analysis. The proposed silver nanoformulation may be used as a next-generation fungicide in crop improvement and may also find application in anticancer investigations. To the best of our knowledge, this is also the first report of silver nanoformulation demonstrating complete inhibition against the economically significant phytopathogen *C. falcatum*.

## Introduction

Fungal diseases pose a significant threat to agricultural crops worldwide, significantly impacting global food production^1^. The enumeration of over 19,000 fungi as causative agents for several economically significant diseases highlights their adverse impact on agricultural crops^2^. Among them, *Sclerotinia sclerotiorum* is a soil-borne and necrotrophic fungus that affects more than 500 plant species, including legumes and oilseeds^3,4^. *S. sclerotiorum* is responsible for white mold or stem rot disease in different crops^5,6^. *Colletotrichum* is positioned within the top ten most devastating phytopathogenic fungal genera on a global scale and members of this genus are hemibiotrophic and cause severe economic losses, especially to vegetables, fruits, and ornamentals^7–9^. *Colletotrichum falcatum* is a soil-borne fungus that causes red rot of sugarcane in tropical and subtropical regions of the world^10,11^. *Colletotrichum gloeosporioides* is a seed-borne fungus that causes anthracnose disease in vegetables and fruit crops^12,13^. *Rhizoctonia solani* (teleomorph *Thanatephorus cucumeris* (Frank) Donk) is a soil- and seed-borne necrotrophic fungus that causes sheath blight disease of rice and affects more than 250 plant species including maize, potato, and soybean^14,15^.

In India, a considerable portion of agricultural yields experience a huge decline primarily because of soil- and seed-borne fungal populations. They play a crucial role in the decomposition of seeds and seed products, rendering them unsuitable for human consumption by compromising their nutritional value through toxin production^16^. There are numerous methods of managing plant fungal diseases viz. synthetic pesticides, biological control, and resistant varieties^17^. The intensive and indiscriminate use of chemical fungicides has resulted in the accumulation of toxic compounds threatening to entire ecosystem^18,19^. The overuse of fungicides has also resulted in the development of resistance among some pathogenic populations^20^. The biocontrol agents, specifically microorganisms coexist in symbiosis with plant roots, however, there are a limited number of studies that investigate their efficacy in inducing resistance against the reported fungi^21,22^.

This necessitates exploring another alternative approach for developing sustainable and effective antifungal products that confer effective control with less harmful effects. Nanotechnology is a developing interdisciplinary research area that offers vital prospects for the development of materials and technology with highly improved and inventive functionalities of nanosized materials due to their increased surface area to volume ratio^23,24^. Nanoparticles are atomic or molecular aggregates with the ability to drastically modify their physiochemical properties compared to their bulk material^25^. Nanoparticles can be made from a variety of bulk materials and can elucidate their actions depending on the chemical composition and size and/or shape of the particles^26^. Among noble metals, silver (Ag) is the preferred nanoparticle because of its broad-spectrum antimicrobial potential^27,28^. Additionally, Ag is also extensively used as a potent therapeutic agent^29^. Silver nanoparticles (AgNPs) exhibit a range of activities, including antibacterial, antifungal, anti-inflammatory, anti-viral, and anticancer properties^30^. Due to their wide antimicrobial properties, AgNPs have been frequently used in different fields including biotechnology, biomedicine, veterinary medicine, pharmacy, food, and electronics with further potential uses in agriculture, plant pathology, ecology, construction, textiles, cosmetics, and other industries^31,32^.

The green synthesis of nanoparticles has received enhanced attention due to its environmentally friendly nature^33,34^. Substantial efforts have been directed towards the biosynthesis of inorganic materials, especially metal nanoparticles, utilizing microorganisms and plants^35,36^. The synthesis of AgNPs mediated by a biological route, i.e., a green process, is considered superior to conventional chemical and physical methods^37^. Traditional physio-chemical techniques involve the use of hazardous chemicals or high energy requirements, which are rather difficult and include wasteful purification^38,39^. Chemical reduction, autoclaving, gamma radiation, and electrochemical processes have previously produced high yields of AgNPs, but they were energy-intensive, high cost, and generated harmful byproducts^40^. The toxic chemicals, capping agents, and reducing agents in these methods have detrimental effects on the environment. Therefore, there is a vital need for an alternative, eco-sustainable approach^41^. Earlier reported literature highlighted green synthesis as a non-toxic, environmentally friendly, cost-effective, and sustainable approach^42,43^. It is a single-step method that can be easily scaled up for large-scale synthesis and does not require high pressure, temperature, energy, or toxic chemicals^44^. Plant extracts are considered to be an excellent and benign source for AgNPs synthesis because the phytochemicals present in plant extracts have the potential to reduce silver ions (Ag^+^) to metallic silver (Ag^0^) in a shorter time compared to algae, bacteria, fungi, and other microbes, which demand a longer incubation time^45,46^. Plant extract plays a crucial role as a reducing, capping, and stabilizing agent to facilitate the synthesis of AgNPs^47^. The plant leaf extract contains secondary compounds that serve as precursor molecules, acting as reducing, capping, and stabilizing agents for nanoparticle synthesis^48^. The nanoparticles synthesized through the green method employing aqueous plant leaf extract are more effective and possess superior antimicrobial properties than those that are produced via thermal, chemical, or other biological routes^41^.

The green synthesized AgNPs are also reported for their anticancer activities^49^. Cancer, being a pervasive global health issue and a leading cause of one in six deaths worldwide, necessitates effective treatment modalities^50^. Although chemotherapy remains a widely employed approach due to its proven efficacy, its associated adverse effects, such as hair loss, fatigue, oral discomfort, and skin problems, present considerable risks^51^. Furthermore, certain modern anticancer agents exhibit limited efficacy, and the widespread use of antibiotics has contributed to the emergence of multidrug-resistant bacteria worldwide^52^. Consequently, there is an utmost need to identify novel compounds with both antimicrobial and anticancer activities. With the remarkable versatility of AgNPs across biomedicine, agriculture, and environmental domains, there is a persistent demand for the development of cost-effective and eco-sustainable methods for AgNPs synthesis^31,53^. The translation of silver-based nanotechnology into agricultural and clinical applications necessitates the non-toxic, simple, and environmentally friendly methods for AgNPs synthesis^54,55^. Moreover, the understanding of in vitro and in vivo effects and safety controls is essential for advancing the field and ensuring the successful integration of silver-based nanotechnology into practical applications in agriculture, healthcare, and related domains^54,56^.

In the present study, we have used the potential inherent in plant-based sources for the synthesis of AgNPs as an eco-friendly alternative to conventional methods^39^. The leaf extract of *Azadirachta indica*, commonly known as neem and belonging to the *Meliaceae* family was utilized for the bioconversion of Ag^+^ ions to Ag^0^, leading to the subsequent synthesis of AgNPs^57^. *A. indica* is widely available in India and has been traditionally utilized for viral, bacterial, and fungal infections^58^. The leaf extract of *A. indica* serves as a non-hazardous reducing agent in the green synthesis of AgNPs. Notably, terpenoids and flavanones, essential phytochemicals present in *A. indica*, play a pivotal role in stabilizing the nanoparticles, functioning as both capping and reducing agents^59^.

The earlier reported methods for the green synthesis of AgNPs were synthesizing nanoparticles in liquid formulations that had high agglomeration rates and used low molarity ranging from 1 to 5 mM^58–63^. Nevertheless, the present study optimized the increased molarity to yield AgNPs in a powdered form^64^. The liquid formulation necessitates extensive processing for characterization, as analytical tools such as Fourier transform infrared spectroscopy (FTIR), X-ray diffractometer (XRD), field emission scanning electron microscope (FESEM), and energy dispersive X-ray analysis (EDX) demand nanoparticles in powdered form for their analysis^65^. Therefore, there has been a long-felt need for an improved method to synthesize nanoparticles in powdered form using a green synthesis approach. The primary objective of the present study was to synthesize AgNPs in powder form with complete solubility and a longer shelf life. The resulting silver nanoformulation was extensively evaluated for its antifungal and cytotoxic activities.

## Material and methods

### Chemicals

Silver nitrate (AgNO_3_) was purchased from Merk, India. Whatman No. 1 filter paper was purchased from Sigma–Aldrich, USA. Sodium hydroxide (NaOH), polyethylene glycol (PEG), potato dextrose agar (PDA), 3-(4,5-dimethylthiazol-2-yl)-2,5-diphenyltetrazolium bromide (MTT), Minimum Essential Medium Eagle (MEM), penicillin–streptomycin and trypsin–EDTA were purchased from Himedia Laboratories Pvt. Ltd., India. Fetal bovine serum (FBS) was purchased from Gibco, USA. All chemicals were used as received without further purification. Double distilled water was used in all experiments.

### Preparation of *A. indica* extract

*Azadirachta indica* leaf extract was used to synthesize AgNPs based on cost-effectiveness, ease of availability, and antimicrobial and medicinal properties^58^. Fresh and healthy leaves of *A. indica* (neem) were collected from the research fields of Chaudhary Charan Singh University, Meerut, Uttar Pradesh, India. The collected leaves were thoroughly washed 3 to 5 times in tap water followed by double distilled water to remove any impurities such as debris, dirt, and particulate matter on the surface of the leaves. The leaves were shade-dried at room temperature for 10 to 15 days and then finely chopped and ground to a fine powder. The cold maceration method was used to prepare the aqueous plant leaf extract. 25 g of powdered leaves were added to 500 ml of double distilled water and kept at 25 ℃ for 24 h. at 250 to 300 rpm for continuous agitation and mixing. The obtained crude extract was filtered three times through Whatman filter paper number 1 to remove particulate matter and to obtain an aqueous solution of leaf extract. The extract was stored at 4 ℃ and further used for the green synthesis of AgNPs. In every step of the experiment, sterility conditions were maintained for the effectiveness and accuracy of the results.

### Green synthesis of AgNPs

50 ml of aqueous leaf extract of *A. indica* was heated at 90 °C on a magnetic stirrer. When the temperature of the extract reached 55 °C, approximately 5 g of AgNO_3_ was added to obtain a 0.6 M AgNO_3_ solution. This reaction mixture was left for approximately 2 to 3 h. 0.2 g NaOH pellet was added slowly to the solution, and the formation of a black precipitate was observed. The precipitate was allowed to settle overnight and washed adequately with double distilled water. The obtained precipitate was centrifuged at 6000 rpm for 10 min. The supernatant was discarded, and the obtained pellet was washed 3 to 4 times with 70% ethanol and left overnight in a hot air oven at 45 to 50 °C. The obtained black powder was the synthesized AgNPs that were stored in an airtight container for further characterization. The obtained powder form of AgNPs was completely dispersed in 5 g/L PEG to develop a silver nanoformulation. The obtained silver nanoformulation was stored at room temperature for 1 year to assess its shelf life and bioavailability. The silver nanoformulation was then used for antifungal and cytotoxic analysis. An Indian Patent Application (Application No.: 202211043204 A) has been filed for the aforementioned^66^.

### Characterization of AgNPs

The optical properties of synthesized AgNPs through the bio-reduction of Ag^+^ using aqueous leaf extract of *A. indica* were estimated through a UV–visible spectrophotometer (Lasany International, F825R12HB). UV–vis spectral analysis was carried out at wavelengths with a resolution of 1 nm within the range of 200–800 nm. The presence of AgNPs is inferred by the observation of a noticeable peak within the wavelength range of 400 to 500 nm^67^. The shape of the AgNPs was determined through FESEM (Carl Zeiss, Ultra Plus series). The samples of the AgNPs were mounted on the grid using carbon or copper tape and sputtered with gold using a sputter coater (Quorum Q150R ES, Quorum Technologies Ltd. Ashford. Kent. England). The voltage was set at 20 kV beam energy to obtain the excitation of all the elements and magnification at 25,000× to 1,00,000×^54^. Analysis of elements was carried out by EDX attached to the FESEM mentioned above at the Institute Instrumentation Center, IIT Roorkee, India. XRD (Bruker AXS, D8 Advance) was used to analyze the crystallite structure and size of the AgNPs. The XRD pattern was recorded by Cu-Kα radiation with about 1.54060 Å (2θ range from 20° to 80°)^68^. FTIR was used to identify the functional groups and various phytochemical constituents involved in the reduction, capping, and stabilization of the synthesized AgNPs in the range of 4000–400 cm^−1^. FTIR was carried out using the attenuated total reflectance (ATR) mode with a PerkinElmer FTIR L1600300 (Llantrisant, U.K.).

### Antifungal analysis of silver nanoformulation

Pure fungal cultures of *S. sclerotiorum* (ITCC No. 447), *C. falcatum* (ITCC No. 4800), *C. gloeosporioides* (ITCC No. 6933), and *R. solani* (ITCC No. 7855) were procured from the Indian Type Culture Collection (ITCC), Division of Plant Pathology, Indian Agriculture Research Institute, New Delhi, India. The morphology of spores of all four phytopathogenic fungi was studied using an inverted phase contrast microscope (ECLIPSE Ts2-FL).

An in vitro analysis was carried out to determine the antifungal activity of the developed silver nanoformulation using the poison food technique against all four phytopathogenic fungi. Different concentrations of silver nanoformulation (10, 25, 50, 100, 250, 500, 1000, and 2000 ppm) were added to sterilized PDA media. The hyphae of the actively growing mycelium of a 5 to 8-day-old fungal culture were placed in the center of the petri dish. Plates with 5 g/L PEG were used as a negative control, and plates with 2 g/L carbendazim and mancozeb were used as a positive control. All petri dishes were incubated for 7 days at 25 ± 2 °C for the antifungal analysis of silver nanoformulation against *S. sclerotiorum* and at 28 ± 2 °C for the antifungal analysis of silver nanoformulation against *C. falcatum*, *C. gloeosporioides*, and *R. solani*. The circular growth of mycelium was measured after 3, 5, and 7 days of incubation. The experiments were performed in triplicate. The percent inhibition of fungi was calculated using the following formula^69^:

$$\mathrm{Inhibition\, of\, mycelium }\left(\mathrm{\%}\right) =\frac{\mathrm{gc }-{\text{gt}}}{{\text{gc}}}\times 100$$

where, gc = negative control plates showing the growth of mycelium and gt = silver nanoformulation treated plates showing the growth of mycelium.

### Cytotoxicity analysis of silver nanoformulation

#### Culture of U87MG glioblastoma multiform cells

The human glioblastoma cell line U87MG was purchased from the National Centre for Cell Science, Pune, India. The cell line was cultured in MEM supplemented with 10% FBS, 100 U/ml penicillin, and 100 g/L streptomycin in a humidified atmosphere with 5% CO_2_ at 37 °C. The culture medium was refreshed at 2-day intervals. Cells were then passaged when they reached approximately 80 to 90% confluence^70^.

#### MTT assay

The MTT dye reduction assay was used to determine the cytotoxic effects of silver nanoformulation on U87MG glioblastoma multiform cells. These cancerous cells (2 × 10^4^ cells/ml) were seeded in 96-well plates, which were cultured for 24 h. Then, 100 μL of media containing different concentrations of silver nanoformulation (10, 20, 30, 50, 100, 150, 200, and 250 ppm) were separately added to the cells. U87MG cells not treated with silver nanoformulation were used as a control group. All treatments were performed in triplicate. After 24 h. of incubation, the viability of the cells was tested using the MTT assay. Then, 100 μL of MTT (5 g/L) was added to each well, and the plates were incubated for 4 h. at 37 °C. The resulting formazan crystals were dissolved in 150 μL of DMSO with gentle shaking at 37 °C for 5 to 10 min. Then, absorbance was measured at 590 nm with an ELISA microplate reader (MicroScan MS5608A, Electronics Corporation of India Limited)^71^. The results were obtained using three independent experiments. The percentage of cell death was calculated using the following formula^72^:

$$\mathrm{Percentage\, of\, cell\, death\, }= 100- ((\mathrm{Absorbance\, of\, sample}/\mathrm{Absorbance\, of\, control}) \times 100)$$

### Statistical analysis

The experimental design utilized in this analysis was a randomized block design, implementing the treatments of silver nanoformulation in triplicate. The statistical analysis was performed using SPSS (Version 25) software and R studio (4.3.1) software, employing the analysis of variance (ANOVA) technique. Specifically, a three-way ANOVA using the general linear model was conducted for the antifungal analysis. One-way ANOVA was employed to compare the means of cell viability for cancerous cells. Means were compared using Tukey’s post hoc test, with the evaluation of significant differences at a significance level of 5%.

The EC_50_ (half maximal effective concentration of the silver nanoformulation required to obtain a 50% antifungal activity against phytopathogenic fungi) and IC_50_ (half minimal inhibitory concentration of the silver nanoformulation required for 50% inhibition against cancerous cells in vitro) values were calculated using the Prism program (GraphPad Prism 10 software) through nonlinear regression analysis^73^.

### Ethical approval

We comply with relevant guidelines and legislation regarding the sample collection and use in the present study. The plant leaf (*Azadirachta indica*), in the present study is not endangered.

## Results

### Green synthesis of AgNPs

**Figure 1:**
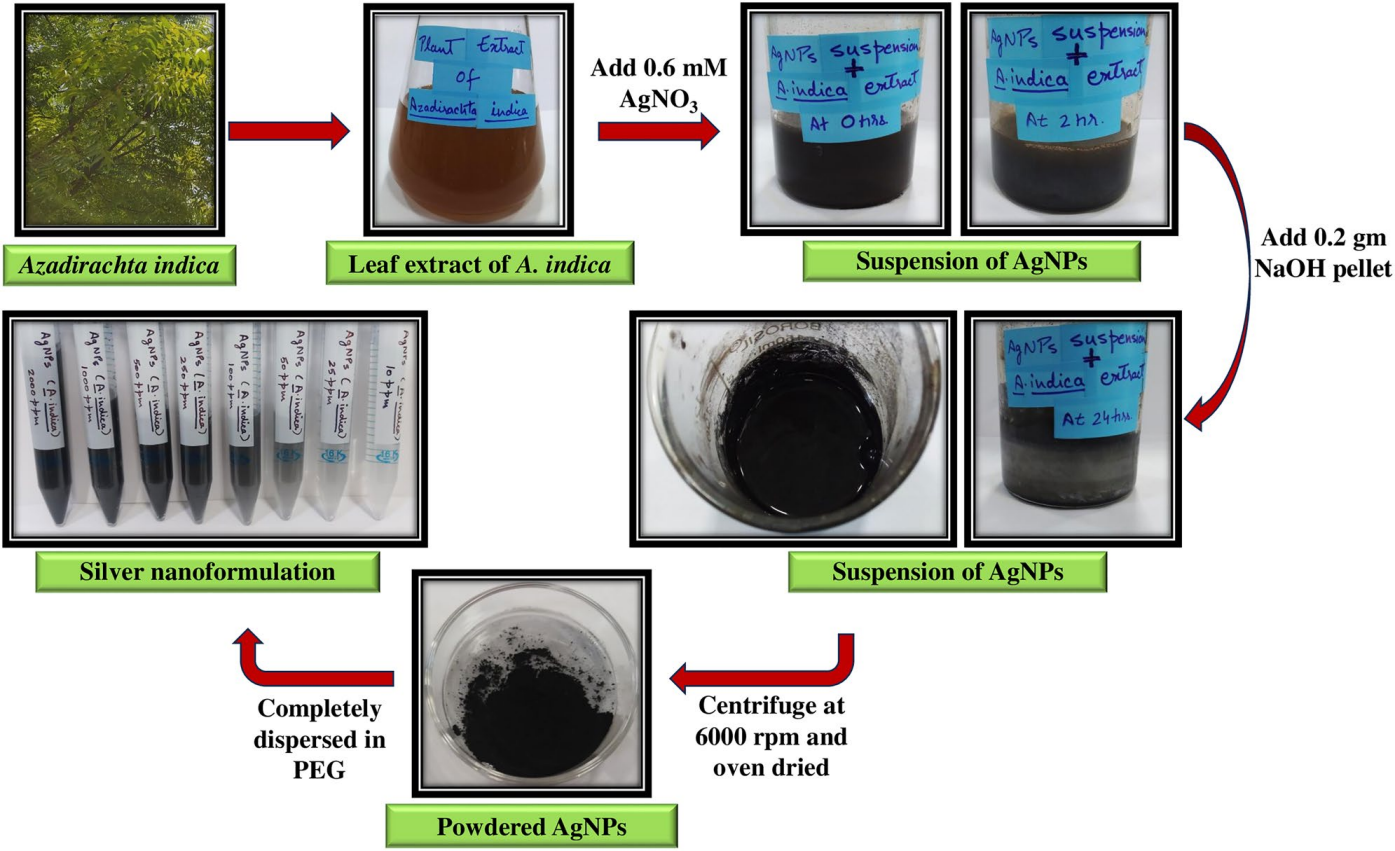
Schematic representation of green synthesis of silver nanoformulation using *A. indica.*

The phytochemicals present in the aqueous leaf extract of *A. indica* when mixed with AgNO_3_, reduced Ag^+^ ions to Ag^0^ and synthesized AgNPs. The organic moieties further capped and stabilized the AgNPs. The synthesis of AgNPs was preliminarily confirmed by the change in color, i.e., from brown to black. The color change indicated the reduction of Ag^+^ ions to Ag^0^ and the synthesis of AgNPs. After centrifugation and overnight heating, the obtained powdered form of AgNPs was completely dispersed in PEG to develop a silver nanoformulation (Fig. 1). PEG enhances the stability of the silver nanoformulation, preventing the aggregation of nanoparticles even when tested after an extended period of more than a year.

### Characterization of AgNPs

#### UV–vis spectrophotometer analysis

**Figure 2:**
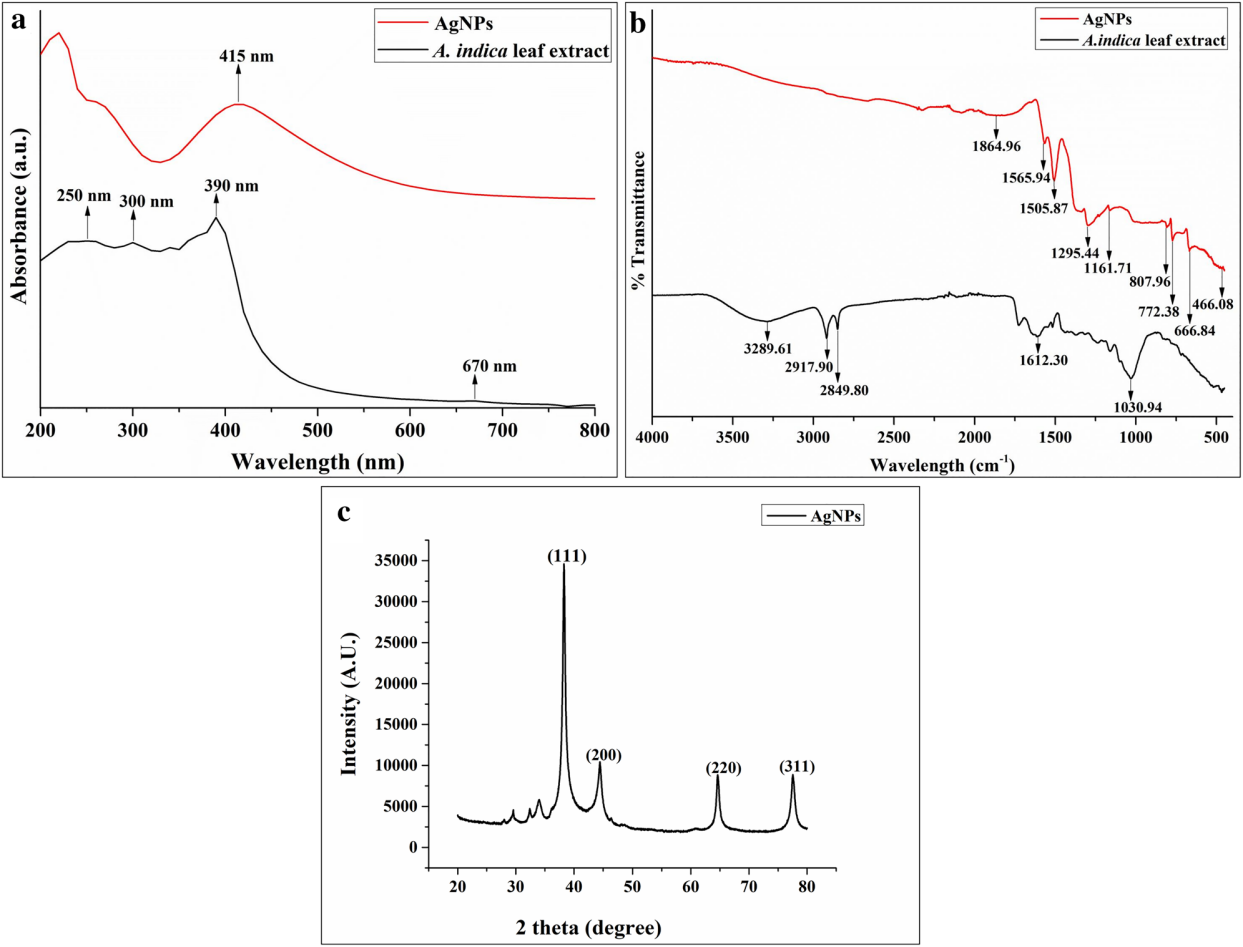
Characterization of synthesized AgNPs (**a**) UV–vis spectrum of AgNPs, (**b**) Fourier transform infrared spectrum of AgNPs, (**c**) X-ray diffraction pattern of AgNPs.

The UV–vis spectrophotometer showed a strong absorption peak at 415 nm (Fig. 2a) due to the mutual vibration of Ag-NPs free electrons in resonance with the light wave^74^ and hence, the bioreduction of Ag^+^ ions into Ag^0^ ions occurred due to the synthesis of AgNPs. The surface plasmon resonance (SPR) values below or higher than 400 nm indicate smaller or bigger nanoparticles, respectively^75^.

#### FTIR analysis

The green synthesized AgNPs have been investigated for the biological moieties for the FTIR measurements to identify the *A. indica* leaf extract’s possible biomolecules and their possible involvement^54^. FTIR spectrum delineated distinctive bands and peaks corresponding to various functional groups linked to the *A. indica* leaf extract, and subsequently to the AgNPs. The alterations in the FTIR spectrum pattern corroborated the association of the *A. indica* leaf extract in the synthesis process of AgNPs. The FTIR spectrum pattern from the *A. indica* leaf extract was observed at 3289.61, 2917.90, 2849.80, 1612.30, and 1030.94 cm^−1^. The strong, broad band at 3289.61 cm^−1^ was due to the O–H stretching of alcohol and carboxylic acid. The intense peak at 2917.90 cm^−1^ was due to the N–H stretching of the amine group. The sharp peak at 2849.80 cm^−1^ was due to the C–H stretching of alkane. The peak at 1612.30 cm^−1^ was either due to the C=C stretching of alkene or the N–H bending of the amine group. The strong peak at 1030.94 cm^−1^ was due to either the C–O stretching of aromatic ester or the S=O stretching of sulphoxide. The AgNPs also showed different bands and peaks at 1864.96, 1565.94, 1505.87, 1295.44, 1161.71, 8087.96, 772.38, 666.84, and 466.08 cm^−1^. The broad band at 1864.96 cm^−1^ was observed due to the bending of the C–H bond of aromatic compounds. The peak at 1565.94 cm^−1^ was observed due to the C=C stretching of cyclic alkenes. An intense peak at 1505.87 cm^−1^ was observed due to the stretching of nitro compounds. The peak at 1295.44 cm^−1^ was observed due to the C–N and C–O stretching of the aromatic amines and aromatic ester, respectively. The peak at 1161.71 cm^−1^ showed the presence of ester and tertiary alcohol due to the C–O stretching. The peak at 807.96 cm^−1^ was observed due to the C=C bending of alkene. Small peaks at 772.38 cm^−1^ and 666.84 cm^−1^ were observed due to the presence of halo compounds, viz., C–Cl and C–Br stretching (Fig. 2b). Hence, the FTIR spectrum of the synthesized AgNPs indicates the shifts in the peaks as compared to *A. indica* and revealed the organic components such as alkene, nitro, amine, aromatic ester and alcohol present in the *A. indica* leaf extract successfully promoted the synthesis of green synthesized AgNPs during the reduction process and adsorbed on metal nanoparticles’ surface. Further, these biomolecules may aid in preventing the AgNPs from aggregating and so maintaining their long-term stability^76^.

#### X-ray diffraction analysis

XRD analysis confirmed the crystalline nature of the synthesized AgNPs. The XRD pattern showed intense diffraction peaks at 2θ = 38.290°, 44.237°, 64.649° and 77.572° corresponding to the silver crystal planes (111), (200), (220) and (311), respectively (Fig. 2c). The peaks match the standard Joint Committee on Powder Diffraction Standards (JCPDS) data file No. 04-0783. The obtained peaks at the 2θ value confirmed that the synthesized AgNPs possess a face-centered cubic (fcc) structure^77^. The average crystallite size of the nanoparticles was calculated from the full-width half maximum and Bragg reflections by using the following Debye–Scherrer equation^78^:

$${\text{D}}=\mathrm{K\lambda }/\mathrm{\beta cos\theta }$$

where D is the crystal size estimated from the XRD patterns, θ is the Bragg angle in degrees, λ is 1.5406 Å, the wavelength of the X-ray source used, β is the full-width half maximum (FWHM) of the diffraction peak in radians and K is the constant (geometric factor) of Debye–Scherrer’s equation. The estimated particle size of the synthesized AgNPs was 8.78 ± 2 nm.

### FESEM and EDX analysis

**Figure 3:**
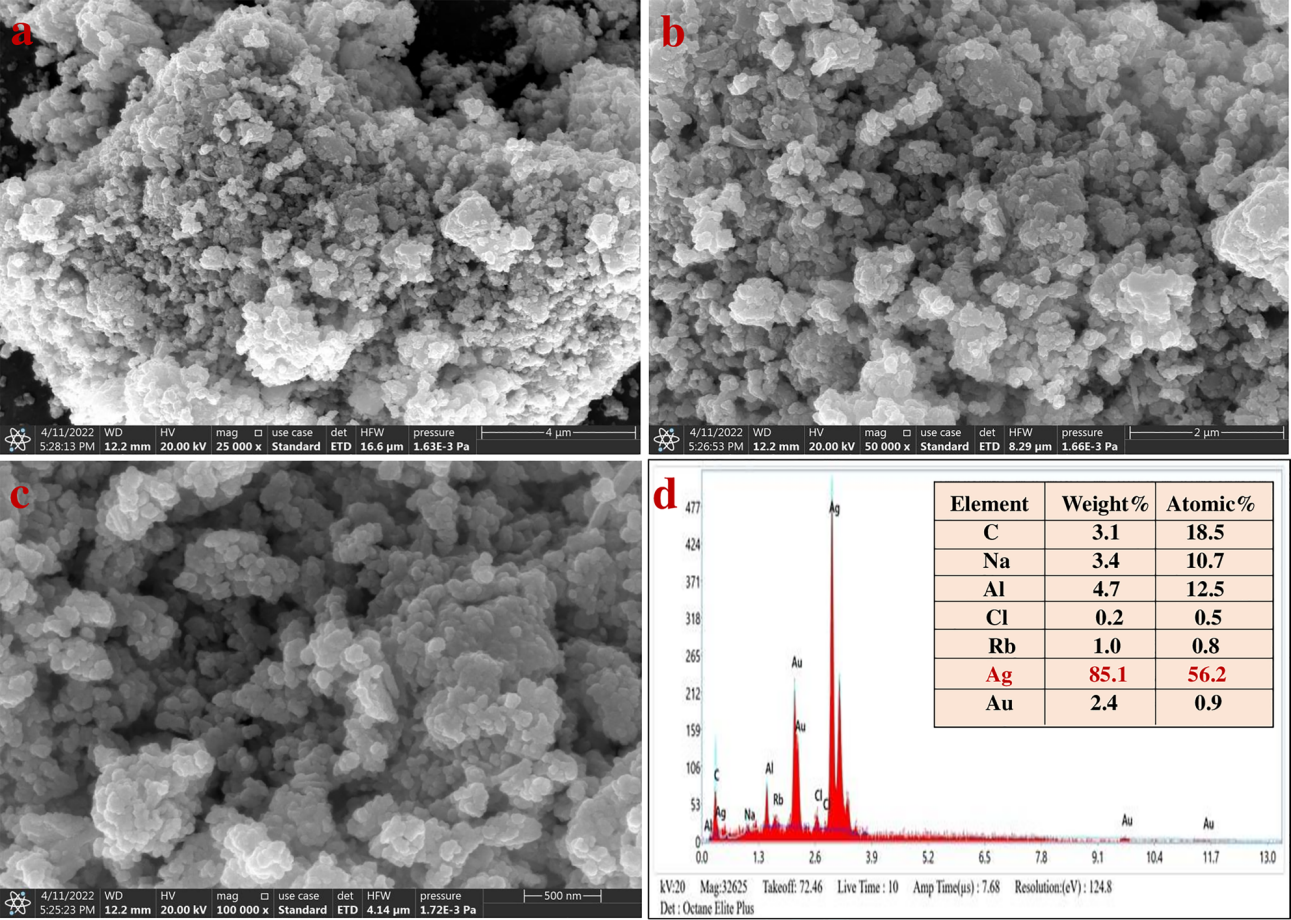
Field emission scanning electron microscope images of synthesized AgNPs (**a**) At 4 µm. (**b**) 2 µm (**c**) 500 nm, (**d**) energy dispersive X-ray analysis of AgNPs.

The morphology and distribution of the synthesized AgNPs were ascertained through FESEM. The FESEM images at 4 µm, 2 µm, and 500 nm scale at 25,000×, 50,000×, and 100,000× magnification respectively showed nanoparticle aggregates that were spherical and polydispersed (Fig. 3a–c). EDX images showed a qualitative and quantitative analysis of the elements present in the synthesized AgNPs^79^. The elemental analysis revealed that Ag was 85.1% at 3 keV. Ag was the major constituent element, while the other reported peaks of carbon (C), chlorine (Cl), gold (Au), aluminium (Al), sodium (Na), and rubidium (Rb) may be due to the carbon-coated copper grid or the emission of X-rays from the capped phytochemicals of plant leaf extract (Fig. 3d)**.**

### Morphological study

**Figure 4:**
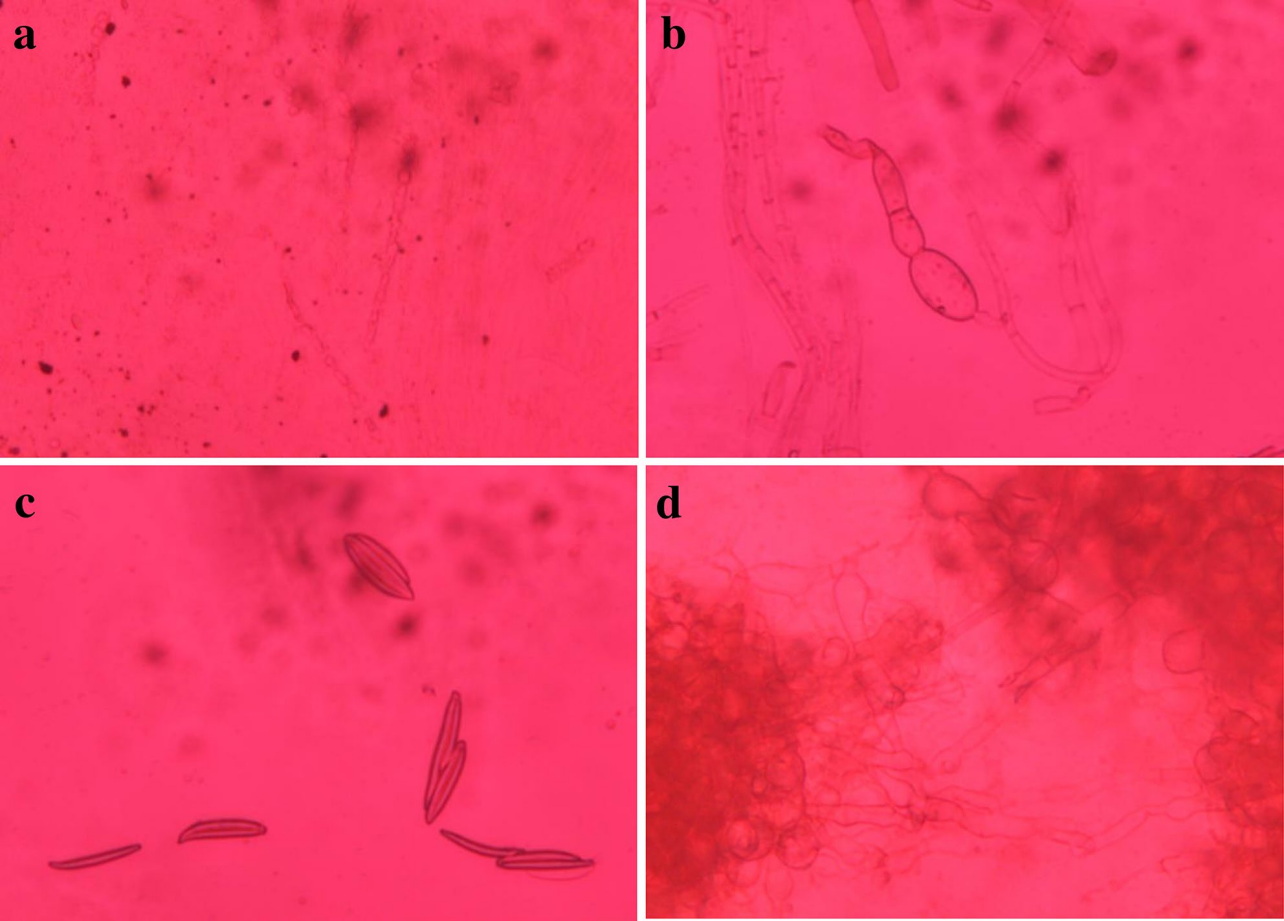
Spore morphology of all four phytopathogenic fungi (**a**) *S. sclerotiorum*, (**b**) *C. falcatum*, (**c**) *C. gloeosporioides*, (**d**) *R. solani.*

The morphology of spores of all four phytopathogenic fungi was established using an inverted phase contrast microscope. Spores of *S. sclerotiorum* showed a dichotomous branching pattern (Fig. 4a), *C. falcatum* showed septate tape-shaped spores with bulbs on one edge of the spores (Fig. 4b), *C. gloeosporioides* showed sickle or canoe shaped spores (Fig. 4c) and *R. solani* showed bulb shaped spores (Fig. 4d).

### Antifungal analysis of silver nanoformulation

**Figure 5:**
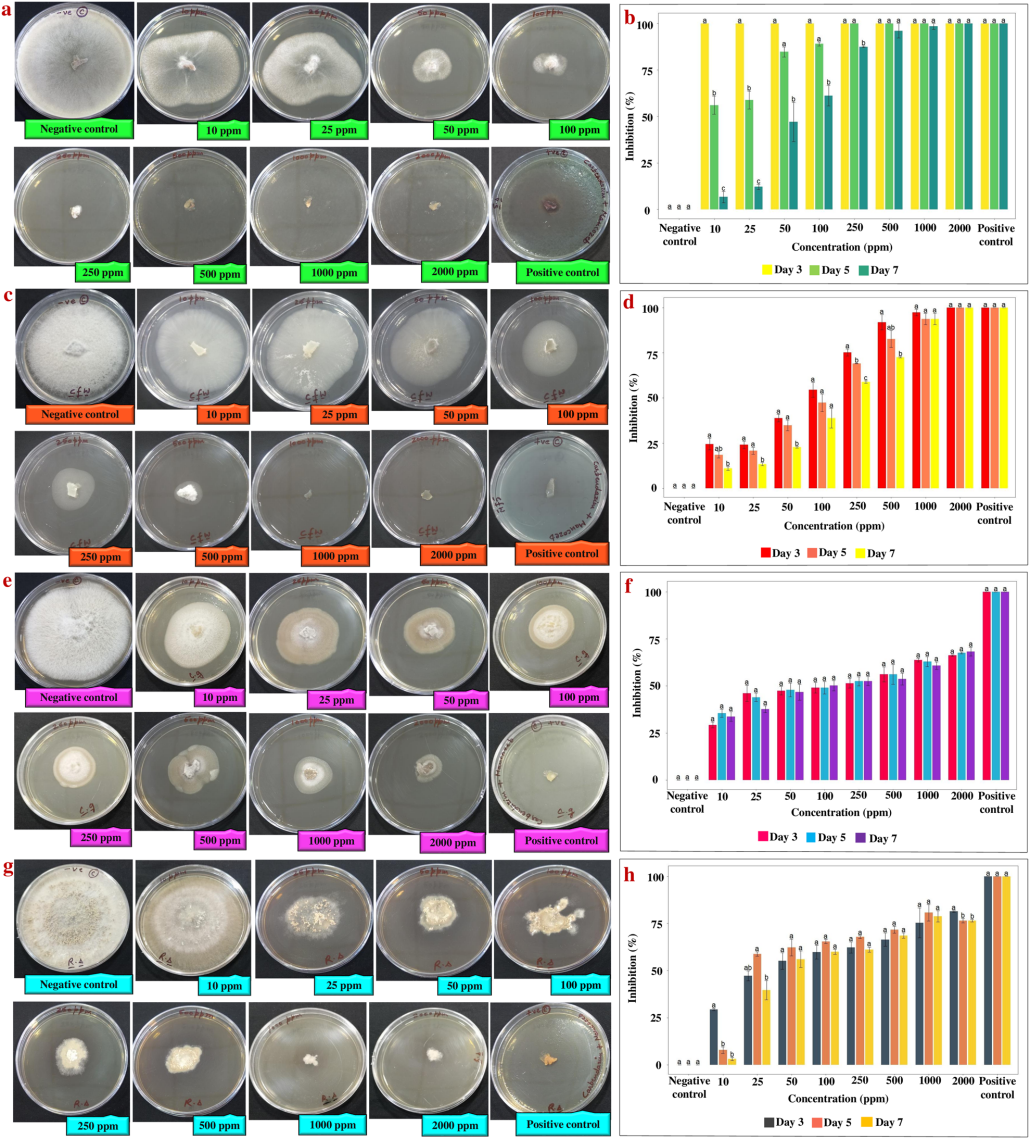
Antifungal analysis showing the zone of inhibition and percentage of inhibition against all four phytopathogenic fungi (**a**,**b**) *S. sclerotiorum*, (**c**,**d**) *C. falcatum*, (**e**,**f**) *C. gloeosporioides*, (**g**,**h**) *R. solani*. The specific concentrations of silver nanoformulation that were significantly different from each other based on their percentages of inhibition are indicated by the compact letter based on Tukey's test. Groups with different letters were significantly different, while groups with the same letter were not significantly different.

**Figure 6:**
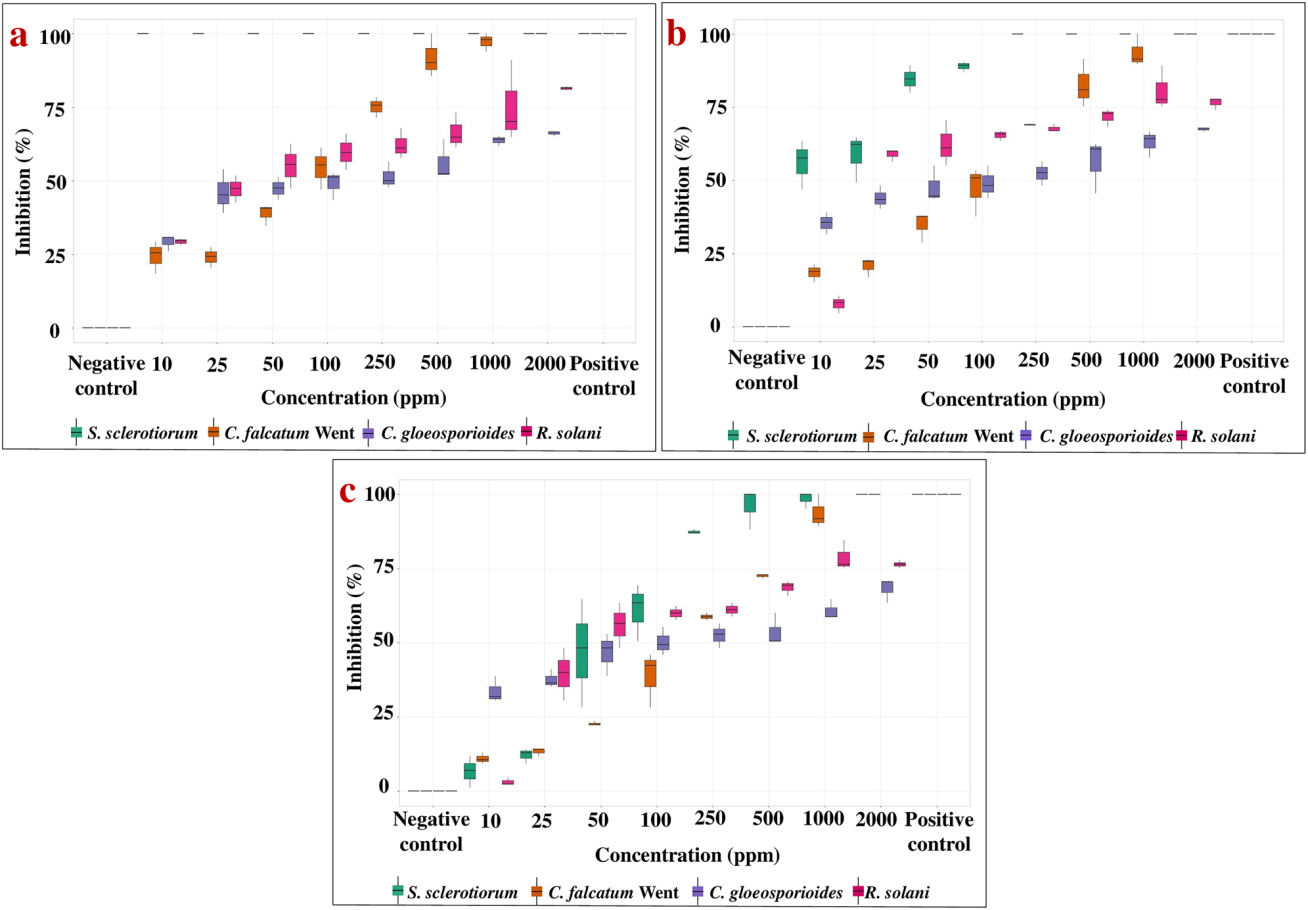
Box plot analysis shows the percentage of inhibition among all four phytopathogenic fungi at different concentrations of silver nanoformulation after different days of inoculation (**a**) Day 3, (**b**) Day 5, (**c**) Day 7.

**Figure 7:**
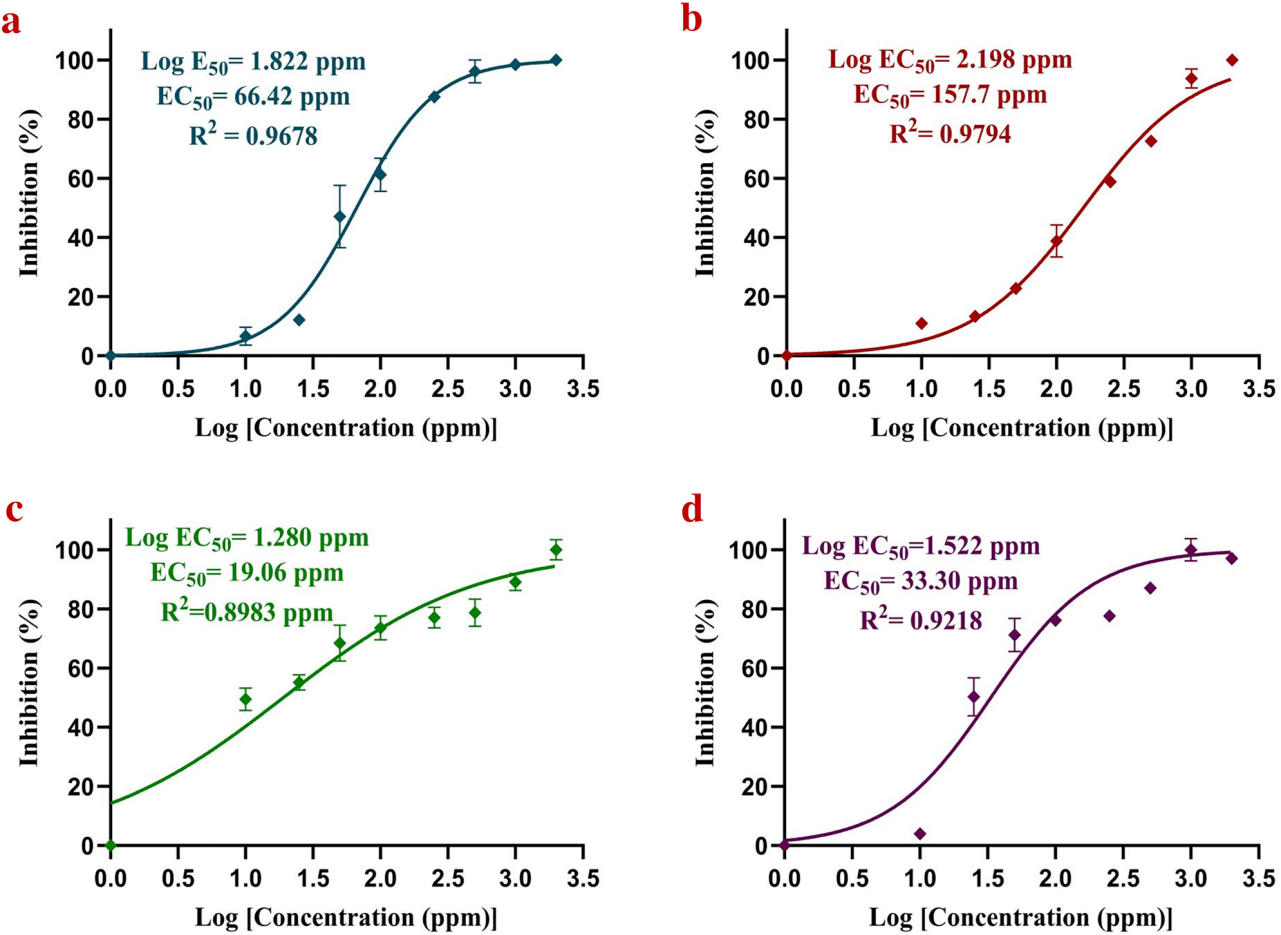
EC_50_ analysis of silver nanoformulation against (**a**) *S. sclerotiorum*, (**b**) *C. falcatum*, (**c**) *C. gloeosporioides*, (**d**) *R. solani*.

The in vitro antifungal activity was assessed by measuring the zone of inhibition and further calculating the percentage of inhibition at different days and different concentrations of silver nanoformulation. The zone of inhibition showed reduced growth of all four fungi with increasing concentrations of silver nanoformulation. The calculated percentage of inhibition of the silver nanoformulation at 10 to 2000 ppm after 7 days of incubation against *S. sclerotiorum*, C*. falcatum*, *C. gloeosporioides* and *R. solani* was 6 to 100%, 10 to 100%, 33 to 68% and 3 to 80% respectively (Fig. 5). Furthermore, evaluation of the percentage of inhibition among all four phytopathogenic fungi at different concentrations and days through box plot analysis illustrated that *S. sclerotiorum* and *C. falcatum* exhibited a higher percentage of inhibition than *C. gloeosporioides* and *R. solani* at different concentrations of silver nanoformulation (Fig. 6). Additionally, the EC_50_ values calculated for all four phytopathogenic fungi were 66.42, 157.7, 19.06 and 33.30 ppm for *S. sclerotiorum*, *C. falcatum*, *C. gloeosporioides* and *R. solani,* respectively (Fig. 7).

### Cytotoxicity analysis of silver nanoformulation

**Figure 8:**
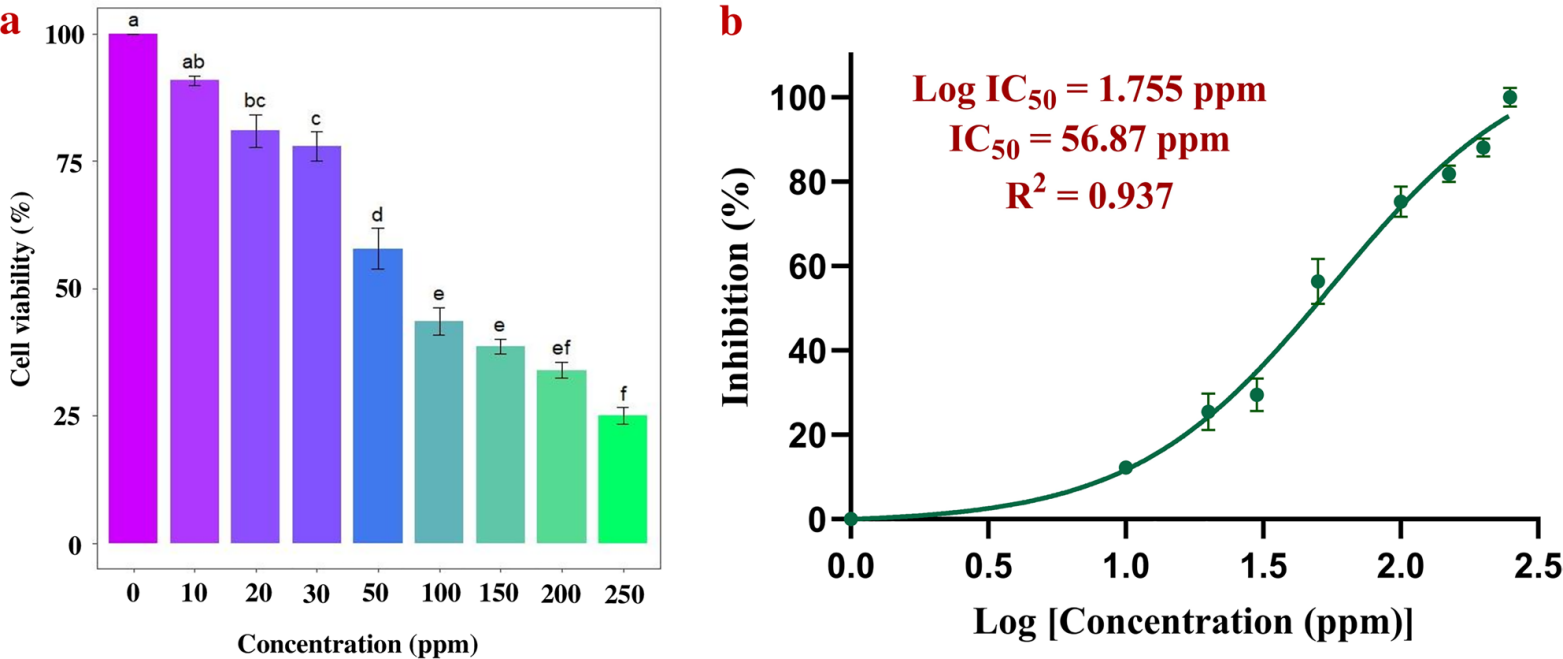
Cytotoxicity analysis against U87MG glioblastoma multiform cell lines (**a**) Bar plot analysis shows the effect of different concentrations of silver nanoformulation on cancer cell viability (error bars indicate standard error). The compact letter based on Tukey's test revealed significant differences between the concentrations, with distinct letters denoting statistically significant variations in cancerous cell viability, (**b**) IC_50_ analysis of silver nanoformulation against the cancerous cells.

MTT assay was utilized to evaluate the toxicity of the silver nanoformulation against U87MG glioblastoma multiform cells. The treatment and incubation of cancer cells for 24 h. with different concentrations of silver nanoformulation resulted in significant inhibition of the growth and proliferation of cancer cells in a dose-dependent manner. Silver nanoformulation reduced the viability of cancer cells due to the loss of their metabolic activities. The viability of cancer cells exhibited a decrease from 98.81 to 25.04%, accompanied by an increase in the percentage of inhibition from 9.19 to 74.96% across silver nanoformulation concentrations ranging from 10 to 250 ppm (Fig. 8a). The IC_50_ value calculated was 56.87 ppm (Fig. 8b).

## Discussion

Green synthesis is an eco-friendly approach that uses plant-derived materials due to the presence of a wide range of phytochemicals that can act as reducing, capping, and stabilizing agents for the synthesis of nanoparticles^56^. In the present research, an aqueous leaf extract of *A. indica* was used for the bioreduction of Ag^+^ to Ag^0^ to synthesize AgNPs^58^. The brown color of the leaf extract was changed to a black color after the synthesis of AgNPs due to the excitation of surface plasmon resonance (SPR). The excitation of SPR was also responsible for the absorption peak of AgNPs at 415 nm in their colloidal solution observed through a UV–vis spectrophotometer. The metal nanoparticles have free electrons, which shows the absorption band of SPR due to the combined vibration of electrons of metal nanoparticles in resonance with light waves^80^. As per earlier reports, the SPR band was dependent on the particle size, dielectric medium, morphology, composition, surface chemistry, refractive index, and surrounding environment of the synthesized AgNPs. Hence, the small and broad absorption peak of the synthesized AgNPs was attributed to their small particle size^65,81^. Similar SPR peaks of AgNPs synthesized using *A. indica* were reported by earlier researchers. Shakeel et al. reported an absorbance peak in the range of 436–446 nm that varied depending upon the variation in the concentration of *A. indica* extract^58^. Similarly, Roy et al. obtained an absorbance peak in the range of 420–450 nm^59^. Asimuddin et al. reported the absorbance peak at ∼410 nm^81^. Alharbi and Alsubhi reported an absorbance peak at 429 nm corresponding to the SPR band^82^. In the present research work, the absorbance peak was centered at 415 nm, which was critical for the wavelength range of 400–500 nm for the green synthesized AgNPs and showed similarity to the earlier reported literature.

FESESM provides the ability to study the morphology of the nanoparticles due to their higher resolution and determine the potential of their application in different fields. The obtained result showed spherical-shaped nanoparticles, which resemble clusters of spherical nanoparticles, with different nanodiameters^83^. The EDX profile illustrates the qualitative and quantitative status of elements present in the synthesized AgNPs^84,85^. The elemental constitution showed a strong Ag signal along with weak signals of other elements (viz., C, Cl, Au, Al, Na, and Rb), which may be due to the biomolecules bound to the surface of the synthesized AgNPs. The major constituent of Ag confirmed the purity of the synthesized AgNPs. C and Au peaks may be due to the same being present in the grids. It has been reported that nanoparticles synthesized using plant extracts are surrounded by a thin layer of organic moieties from the leaf extract and thus provide stability to the synthesized AgNPs. This was a major advantage of nanoparticles synthesized using plant extracts over those synthesized using chemical methods^63^.

X-ray diffraction elucidates the atomic arrangement, lattice parameters, and crystal size of the synthesized AgNPs^86^. The XRD pattern showed four peaks of varying intensities for the synthesized AgNPs. The obtained diffraction angles, along with their associated crystallographic planes, confirmed the crystalline behaviour of AgNPs, exhibiting consistency with the JCPDS (04-0783). The crystallite nature and small size of the synthesized AgNPs exhibit consistent alignment with the results of UV–vis spectroscopy.

FTIR spectroscopy identifies various functional groups in the phytoconstituent of *A. indica* leaf extract that are responsible for the bioreduction of Ag^+^ ions and further capping and stabilization of AgNPs. The observed bands and peaks were compared with standard values to identify the relevant functional groups^87^. The obtained FTIR spectrum revealed the presence of various functional groups, viz., alkene, nitro, amine, aromatic ester, and alcohol, on the surface of the synthesized AgNPs. These functional groups were mainly attributed to flavonoids and terpenoids present in *A. indica* leaf extract^88^. The relevant functional groups of these flavonoids and terpenoids were responsible for the efficient reduction of AgNO_3_ into AgNPs and further stabilization and capping of the synthesized AgNPs^57^. The possible mechanism of such chemical reactions involves functional groups such as organic molecules (–OH, –COOH groups) that interact with AgNO_3_. Since AgNO_3_ is a metal salt, it breaks up to give two ions, i.e., Ag^+^ and NO_3_^–^ ions, when dissolved in water. Since OH and COOH groups are acidic, they furnish H^+^ and acquire a negative charge. Negative functional groups, such as O^−^ in alcohols and COO^−^ in acids, are present in *A. indica* leaf extracts and form electrostatic linkages with Ag^+^. During the formation of such types of linkages, Ag^+^ ions are reduced while NO_3_^−^, accepts H^+^ from the OH of tertiary alcohols or from the COOH of acids to form nitric acid (HNO_3_). Since HNO_3_ is water soluble, it remains in the aqueous phase, whereas Ag survives in a free metallic state (Ag^0^) to form AgNPs^89^.

The obtained powder form of synthesized AgNPs was completely dispersed in PEG. It increases the shelf life and bioavailability of the synthesized AgNPs in the form of silver nanoformulation by providing colloidal stability to the synthesized AgNPs without any aggregation. The in vitro antifungal analysis illustrates significantly high antifungal activity for all the tested phytopathogenic fungi. The zone of inhibition and the percentage of inhibition were positively correlated with an increased concentration of silver nanoformulation. The AgNPs illustrated a significant antifungal activity and complete inhibition was observed for the mycelium of *S. sclerotiorum* and *C. falcatum* at a concentration of 2000 ppm. However, *C. gloeosporioides* showed ~ 68% *gloeosporioides* antifungal activity at 2000 ppm, while *R. solani* showed antifungal activity of ~ 80% at 1000 and 2000 ppm. The box plot demonstrated an upward trend in the median values (represented by the horizontal line within each box) and the percentage of inhibition among all four phytopathogenic fungi varied significantly across different concentrations. *S. sclerotiorum* and *C. falcatum* exhibited a higher percentage of inhibition than *C. gloeosporioides* and *R. solani* against different concentrations of silver nanoformulation. Remarkably, the highest calculated value of EC_50_ i.e., 157.7 ppm was against *C. falcatum* while the lowest EC_50_ value of 19.06 ppm was against *C. gloeosporioides*. The variation in the antifungal activity for different fungal species might be due to the presence of some resistance mechanism against the silver nanoformulation, and the obtained differences in the percentage of inhibition suggest that not all biological systems exhibit similar behaviour under the influence of the same external agent^90^.

**Figure 9:**
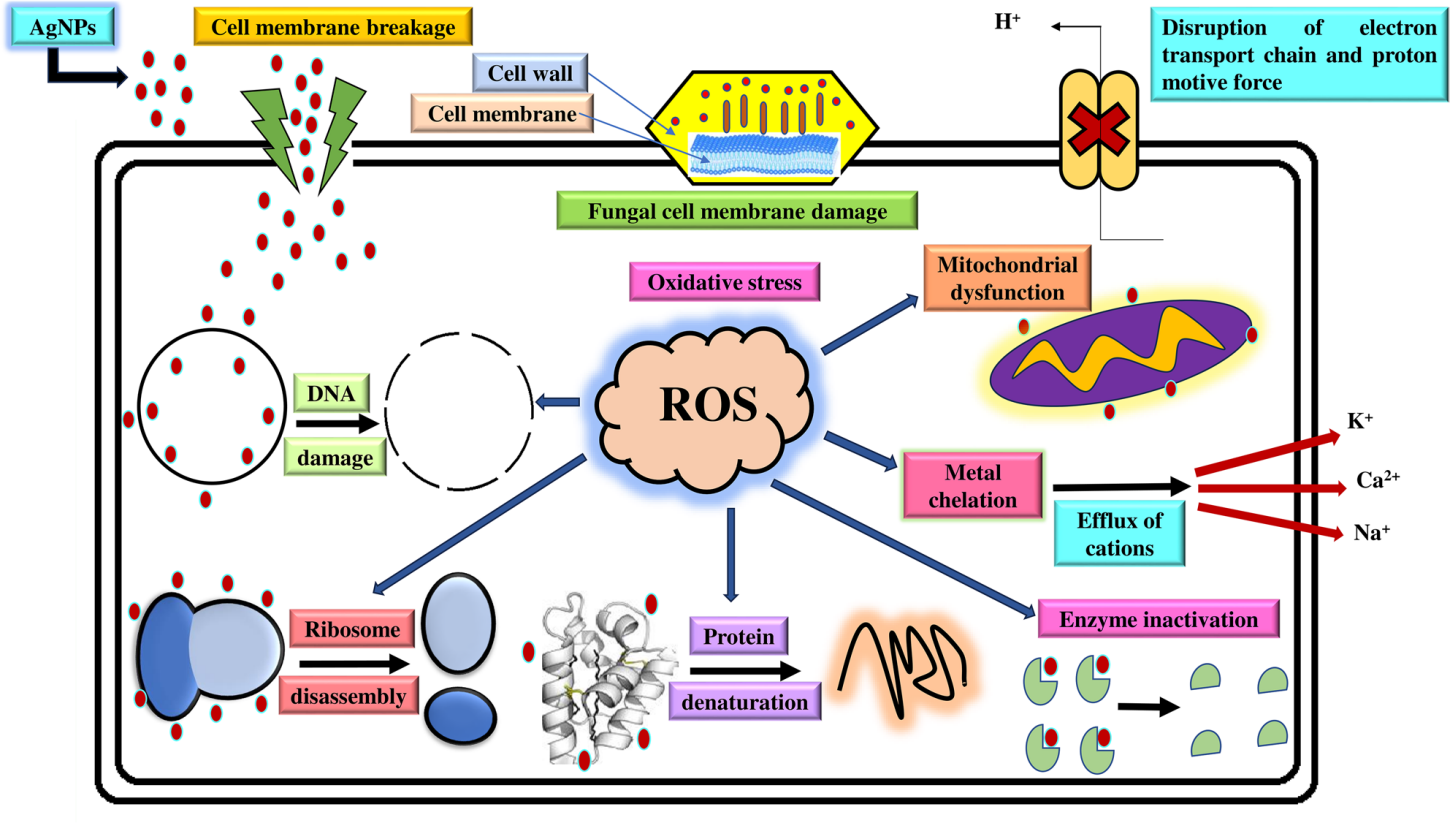
Schematic representation of the plausible mechanism of antifungal activity against the phytopathogenic fungus.

Earlier studies suggested the probable mechanism underlying the antimicrobial effectiveness of AgNPs was attributed to the generation of free radicals and reactive oxygen species (ROS)^91^. These free radicals diminish the activity of antioxidant mechanisms and oxidative enzymes, disrupting osmotic balance and cellular integrity^92^. In the presence of the silver nanoformulation, AgNPs interacting with fungal mycelium led to harm to the fungal cell membrane through the generation of free radicals and ROS^16^. This results in protein denaturation, damage to nucleic acids and proton pumps, lipid peroxidation, and impairment of the cell wall^93,94^. AgNPs enhanced protein and sugar leakage by elevating the membrane permeability of the fungal mycelium, causing cell death^95^. AgNPs accumulate Ag^+^ and block respiration by efflux of intracellular ions and block the proton pump, which leads to mitochondrial dysfunction and induces apoptosis of fungal cells^96^ (Fig. 9).

The cytotoxicity of the silver nanoformulation against U87MG glioblastoma multiform cell lines was also studied to determine its application in the medical field. Cancer is a malignant case of uncontrolled cell proliferation^97^. Among the different cancer types, glioblastoma multiforme is the most common malignant, primary brain tumour, which is alarming and associated with the greatest mortality^98^. Cytotoxicity assays provide information about the reaction of cells to toxic substances, including their survival, death, and metabolism^99^. The MTT assay revealed a negative correlation between the concentration of silver nanoformulation and the viability of cancerous cells, i.e., with increasing concentrations of silver nanoformulation, the viability of cancerous cells decreased. Cells treated with silver nanoformulation showed reduced metabolic activity due to the penetration of AgNPs, which led to severe damage to cancerous cells^100^. The bar plot illustrates the effect of different concentrations of silver nanoformulation at 10 to 250 ppm on cancerous cell viability. The bar heights represent the reduced cancerous cell viability for different concentrations of silver nanoformulation, with the highest inhibition of 74.96% at 250 ppm (the error bars indicate standard error for three replicates). The calculated IC_50_ value against the cancerous cells was significantly low at 56.87 ppm due to lower cell viability of cancerous cell as a result of enhanced cytotoxic activity of silver nanoformulation.

**Figure 10:**
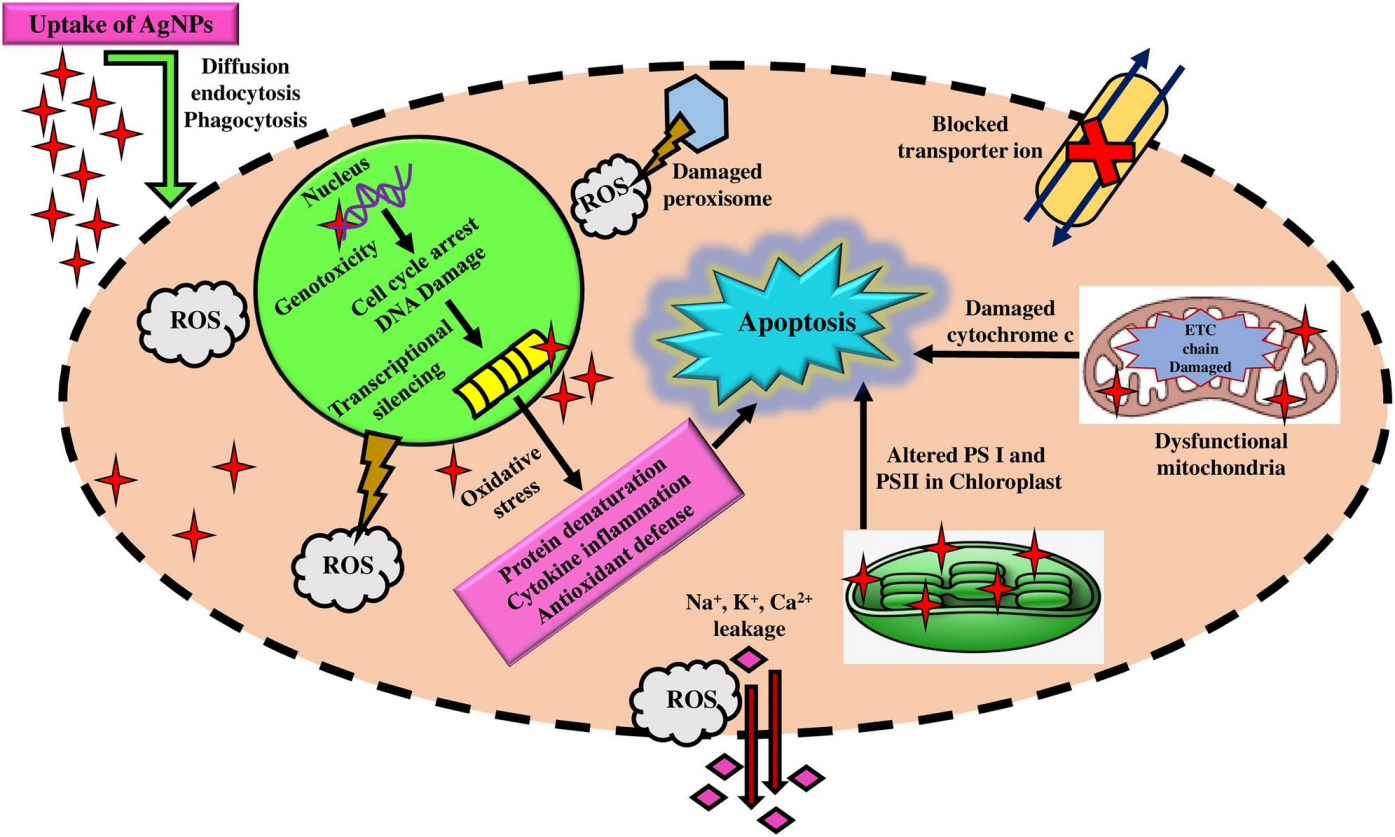
Schematic representation of the plausible mechanism of cytotoxic activity against cancerous cells.

As per the previous studies, the potential mechanisms underlying the cytotoxic effects of AgNPs on cancer cells involve several pathways viz., the induction of oxidative stress through the production of reactive oxygen species (ROS), impairment of mitochondria and DNA, activation of the immune system, and the initiation of cell cycle arrest, ultimately culminating in the apoptosis of cancer cells^101^. AgNPs can permeate cancer cells through processes such as diffusion, endocytosis, and phagocytosis. Furthermore, reports are indicating that the interplay between the positively charged surface of AgNPs and the negatively charged membrane of cancer cells results in membrane rupture. It may promote the influx of ROS into the cancer cell, initiating oxidative stress and ultimately culminating in cell death^102^. AgNPs could also interfere in mitochondrial function and promote ROS production and Ag^+^ ion release inside the cytoplasm of the cancer cells^103^. The excess production of ROS and Ag^+^ ions released from AgNPs can penetrate the nuclear membrane and cause irreversible DNA damage via extrinsic necrosis and apoptosis in a dose-dependent manner in the cancer cell lines^104^. Further, the overproduction of ROS leads to induced oxidative DNA damage and mitotic death and can also trigger autophagic/mitophagic cell death^105^ (Fig. 10). Wang et al. (2021) reported that AgNPs, due to their size and surface charges, exhibit effective cytotoxicity against cancerous cells^102^. Locatelli et al. (2014) reported a cytotoxic effect due to the synergistic activity of multifunctional nanocomposites formed by the drug alisertib and silver against a human glioblastoma–astrocytoma epithelial-like cell line (U87MG)^106,107^. Simsek et al. reported the antiproliferative and apoptotic effects of green synthesized AgNPs using *Lavandula angustifolia* on human glioblastoma cells (U87MG) and obtained a statistically significant dose-dependent decrease in proliferation and increased cytotoxicity in U87MG cells. They also reported an IC_50_ value of 7.536 µg/ml^107^. Alharbi and Alsubhi examined green synthesized AgNPs using the fruit extract of *A. indica*, and AgNPs with cisplatin (AgNP-cis) against the A549 lung cancer cell line and reported cytotoxic and apoptotic effects in vitro. They further suggested AgNPs as good candidates for cancer treatment^83^. However, further investigations are required to examine the toxicity of AgNPs toward healthy cells before their further application as an anticancer drug. Nonetheless, green synthesized AgNPs are less toxic than chemically and physically synthesized AgNPs^108^. Singh et al. showed that AgNPs synthesized from papaya leaf extract had a deficient level of toxicity against normal HaCaT cells^109^. Farmahini Farahani et al. reported less toxicity of phyto-synthesized AgNPs using *Amigdalus spinosissima* extract against normal L929 cells^110^.

The present research successfully illustrated the high efficacy of AgNPs synthesized via the green route against various phytopathogens as well as their application against cancerous cells. It possesses unique physiochemical properties with a long shelf life and bioavailability.

## Conclusion

This study successfully achieved the green synthesis of AgNPs in powder form, utilizing *A. indica* leaf extract. The synthesized AgNPs were in crystalline nature and showed spherical morphology with absolute polydispersity in PEG, making it most suitable for long-term stability. Remarkably, the silver nanoformulation demonstrated complete inhibition against economically important fungi, viz., *S. sclerotiorum* and *C. falcatum,* and significant inhibition against *C. gloeosporioides* and *R. solani*. An additional application of cytotoxicity has also been demonstrated in human glioblastoma cell lines that may further orient its application in anticancer studies. The reported methodology for the green synthesized AgNPs is simple, cost-effective, and user-friendly with minimum environmental hazards. The application of PEG for the complete dispersion of AgNPs for their longer shelf life, stability, and bioavailability makes it more viable for the end user. This is also the first experimental in vitro demonstration of complete inhibition to *C. falcatum*, which is one of the most agriculturally important fungi, causing red rot of sugarcane in India. In the near future, the reported silver nanoformulation can be established as a next-generation fungicide.

## Data Availability

The datasets generated during the current study are available from the corresponding author on reasonable request.
